# Transcription Factors in Biocontrol Fungi

**DOI:** 10.3390/jof11030223

**Published:** 2025-03-15

**Authors:** Han-Jian Song, Xiao-Feng Li, Xin-Ran Pei, Zhan-Bin Sun, Han-Xu Pan

**Affiliations:** School of Light Industry Science and Engineering, Beijing Technology and Business University, Beijing 100048, China

**Keywords:** transcription factor, biocontrol fungi, *Beauveria bassiana*, *Metarhizium*, *Trichoderma*

## Abstract

Transcription factors are extensively found in fungi and are involved in the regulation of multiple biological processes, including growth, development, conidiation, morphology, stresses tolerance, and virulence, as well as the production of secondary metabolites. Biocontrol is a complex biological process through which several biocontrol behaviors, such as the secretion of cell wall-degrading enzymes and the production of secondary metabolites, are regulated by transcription factors. To date, biocontrol-related transcription factors have been reported in several biocontrol fungi, such as *Beauveria bassiana*, *Clonostachys rosea*, *Coniothyrium minitans*, and different species in the genera *Metarhizium*, *Trichoderma*, and *Arthrobotrys*. However, comprehensive reviews summarizing and analyzing transcription factors with biocontrol potential in these fungi are scarce. This review begins by giving a basic overview of transcription factors and their functions. Then, the role of biocontrol-related transcription factors in biocontrol fungi is discussed. Lastly, possible approaches for further work on transcription factors in biocontrol fungi are suggested. This review provides a basis for further elaborating the molecular mechanisms of transcription factors in the context of biocontrol.

## 1. Introduction

Biological control (biocontrol) generally refers to the usage of beneficial organisms—such as fungi and bacteria—to control pathogens, thereby lowering the incidence of plant diseases [1]. Biocontrol has the advantages of being safe, sustainable, and environmentally friendly, which has attracted significant attention. As important examples of biocontrol organisms, biocontrol fungi play crucial roles in controlling plant diseases.

There are a number of diverse mechanisms underlying the control of plant diseases by biocontrol fungi. Biocontrol agents can secrete cell wall-degrading enzymes, including chitinases, glucanases, and some proteases, to degrade the cell walls of plant pathogens [2]. Biocontrol agents can also produce antibiotics or toxins to inhibit the growth of plant pathogens or even kill them [3]. Some fungal mycoparasites can also coil around plant pathogens through a mycoparasitism mechanism to control plant diseases [4]. These behaviors in biocontrol fungi are regulated by the expression activities of transcription factors through upstream signal-transduction pathways, such as the MAPK and cAMP pathways. Deletion of MAPK encoding genes could influence the biocontrol activity of *Trichoderma brevicrassum* in disease control and also affect the expression of biocontrol-related genes, such as genes encoding fungal cell wall-degrading enzymes and genes involved in secondary metabolism [5]. Similarly, disruption of an adenylate cyclase (an important component of the cAMP pathway) encoding gene *crac* could reduce the ability of *Clonostachys rosea* to control plant disease, and the expression of genes encoding cell wall-degrading enzymes were also influenced after *crac* deletion in *Clonostachys rosea* [6]. During the process of biocontrol fungi against plant pathogens, biocontrol-related signals are transmitted through signal-transduction pathways, affecting the expression of transcription factors and, finally, regulating the expression of biocontrol-related genes in biocontrol fungi, thereby controlling plant pathogens. Therefore, transcription factors in biocontrol fungi play important roles in controlling plant diseases.

In this review, the roles and applications of transcription factors in the processes of biocontrol fungi-controlling plant diseases are detailed and discussed. This review demonstrates the importance of understanding the regulatory mechanisms underlying transcription factors in biocontrol fungi.

## 2. Transcription Factors

Transcription factors, commonly known as trans-acting factors, are a type of protein that can specifically interact with related cis-acting elements. Through binding to cis-acting elements, transcription factors can regulate (i.e., activate or inhibit) the expression of downstream genes with specific intensity under specific conditions or circumstances. Common transcription factors contain four functional domains: DNA-binding domain, transcription regulation domain, oligomerization site, and nuclear localization signal [7].

To date, 61 transcription factor families containing 123,899 transcription factors, identified from 61 fungal and three oomycete species, have been reported through fungal transcription factor databases (http://ftfd.snu.ac.kr/index.php?a=view). The bZIP, C2H2 zinc finger, Myb, Forkhead, GATA-type zinc finger, Heteromeric CCAAT factors, Homeodomain-like, Winged helix repressor DNA-binding, Zinc finger, CCHC-type, HMG, and Homeobox families have reportedly had the highest number of transcription factors (262) identified in fungi. The Zn_2_Cys_6_ and Zinc finger, CCHC-type transcription factor families have the highest numbers of transcription factors, 29,247 in fungal species and 966 in oomycete species, out of all transcription factor families.

The Zn_2_Cys_6_ transcription factor contains a DNA-binding domain consisting of six cysteine combined with two zinc. The Zn_2_Cys_6_ transcription factor family is found widely in fungi and is involved in the regulation of multiple biological processes, including growth, development, virulence, secondary metabolite production, nutrient utilization, stress response, and drug resistance. In the *Valsa pyri* mutant, the deletion of the Zn_2_Cys_6_ transcription factor gene *VpxlnR* reduced growth and virulence and caused a loss of fruiting body formation function, and led to higher susceptibility to hydrogen peroxide and salicylic acid, compared with the wild-type strain [8]. In *Fusarium pseudograminearum*, the knockout of the Zn_2_Cys_6_ transcription factor gene Fp487 significantly reduced conidiogenesis, pathogenicity, and 3-acetyl-deoxynivalenol production, and increased sensitivity to oxidative and cytomembrane stress in the mutant [9]. A mutant of *Sclerotinia sclerotiorum* with SsZNC1 gene deletion in the Zn_2_Cys_6_ transcription factor exhibited reduced sclerotial development ability, growth ability, and virulence compared with the wild-type strain [10]. The Zn_2_Cys_6_ transcription factor family is also important in nutrient utilization and drug resistance. The deletion of the Zn2Cys6 transcription factor gene *clr-5* could significantly influence the growth of *Neurospora crassa* through use of leucine or histidine as the sole nitrogen source [11]. The knockout of the Zn_2_Cys_6_ transcription factor gene in *Magnaporthe oryzae* could influence resistance to isoprothiolane [12].

## 3. Functions of Transcription Factors in Biocontrol Fungi

**Figure 1:**
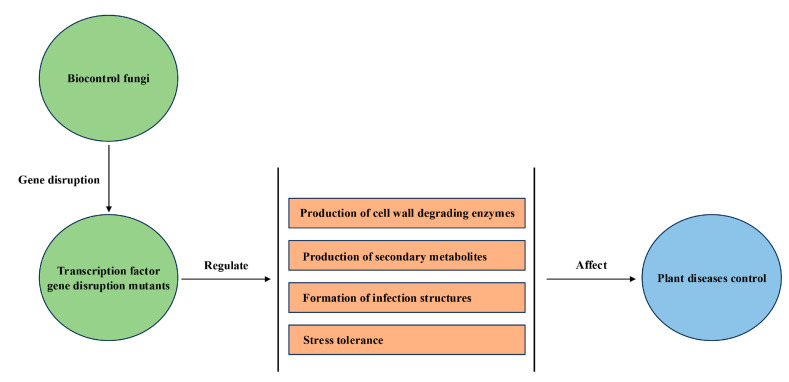
Mechanisms of transcription factors regulate plant diseases control in biocontrol fungi.

Transcription factors are found widely in fungi and have been reported to be involved in the regulation of multiple important biological processes, including fungal growth, development, secondary metabolite production, virulence, and tolerance to environmental stress [13,14,15]. During the process of biocontrol, transcription factors can influence the biocontrol efficiency of biocontrol fungi through regulating the formation of infection structures and stress tolerance, producing cell well-degrading enzymes and secondary metabolites, and participating in other biocontrol behaviors (Figure 1). Hence, transcription factors play crucial roles in biocontrol fungi. To date, transcription factors have been reported as being involved in biocontrol processes in a number of fungal species, such as *Beauveria bassiana*, *Metarhizium* sp., *Coniothyrium minitans*, *Clonostachys rosea*, *Trichoderma* sp., *Hirsutella minnesotensis*, *Arthrobotrys* sp., *Drechslerella dactyloides*, *Paecilomyces lilacinus*, *Purpureocillium lilacinum*, and *Papiliotrema terrestris*. Among these biocontrol fungi, *Beauveria bassiana* and *Metarhizium* sp. (*M. acridum*, *M. robertsii*, and *M. rileyi*) have the most biocontrol-related transcription factors (Table 1).

### 3.1. Beauveria bassiana

*Beauveria bassiana* is wildly used in microbial insecticides to control insect pests in agriculture. Many commercial products have been developed to control a range of insect pests worldwide [16,17]. Multiple transcription factors belonging to different families, including bZIP, Zn_2_Cys_6_, MADS-box, NDT80, p53-like, GATA-type, HSF-type, Homeobox, and C_2_H_2_-type, have been reported to be involved in the biocontrol of insect pests in *B. bassiana*.

Transcription factors might regulate the virulence to insect pests associated with conidial quality, cell wall integrity, oxidative stress response, hyphal body production, appressorium formation, cuticle-degrading proteases, extracellular chitinase, and lipolytic activity.

Transcription factors from the Zn(II)_2_Cys_6_ family are most reported as being involved in virulence in *Beauveria bassiana*. A Zn(II)_2_Cys_6_ transcription factor-encoding gene *BbTpc1* in *B. bassiana* ARSEF 2860 was deleted, and the mutant exhibited reduced virulence to *Galleria mellonella*. The decrease in virulence in the *BbTpc1*-deletion mutant is mainly due to defects in cell wall integrity, as well as growth and resistance, which are important in *B. bassiana* infection [18]. In *B. bassiana* Bb0062, two Zn(II)_2_Cys_6_ transcription factors encoding genes *Bbotf1* and *Thm1* were positively related to virulence. After disruption to *Bbotf1* in *B. bassiana*, conidia recovered from cadavers killed by the disruption mutant showed obviously impaired virulence to *G. mellonella* compared with wild-type and complemented strains. Moreover, *Bbotf1* could also influence resistance to oxidants in *B. bassiana* [19]. The *BbThm1* mutant of *B. bassiana* reduced virulence to *G. mellonella* compared with the wild-type strain. Deep study found that *BbThm1* deletion can influence hyphal body formation and host immune prophenol oxidase response activity in *B. bassiana*, which may result in decreased virulence in the *BbThm1*-deletion mutant [20].

Except for positivity related to virulence, two Zn_2_Cys_6_ transcription factors encoding genes *BbCDR1* and *NirA1* negatively regulate the virulence of *Beauveria bassiana* to insect pests. Disruption to *BbCDR1* in *B. bassiana* CGMCC7.34 can improve virulence to *G. mellonella* and increase yield in conidia. Interestingly, *BbCDR1* can impact conidial development, cell wall integrity, and trehalose synthesis in conidia [21]. Similarly, the *NirA1*-deletion mutant of *B. bassiana* Bb0062 increases virulence to *G. mellonella* [22].

Several transcription factors from the C_2_H_2_-type family, including *BbKlf1*, *crz1*, and *zafa*, have also been reported to be involved in *Beauveria bassiana* virulence. The deletion of *BbKlf1* in *B. bassiana* ARSEF 2860 reduced virulence to *G. mellonella*. The reason for attenuated virulence in the *BbKlf1*-deletion mutant might be due to the secretion of cuticle-degrading Pr1 proteases in the mutant being reduced. In addition, the hyphal bodies were delayed in the *BbKlf1*-deletion mutant compared with the wild type [23]. The *crz1*-deletion mutant of *B. bassiana* ARSEF 2860 remarkably reduced virulence to *Spodoptera litura* compared with the wild-type strain. Conidiation and tolerance to stresses of *B. bassiana* were also influenced by the deletion of *crz1* [24]. Disruption to *zafa* in *B. bassiana* Q2505 could influence growth, spore germination, and sensitivity to stresses, as well as remarkably reduce virulence to *G. mellonella* [25].

Except for positivity related to virulence, a C_2_H_2_-type transcription factor-encoding gene *Bbsmr1* in *Beauveria bassiana* ARSEF 2860 negatively regulated virulence to insect pests. Disruption to *Bbsmr1* could significantly improve virulence to *G. mellonella* compared with the wild-type strain. The increased virulence of the *Bbsmr1* disruption mutant may be due to *Bbsmr1* being related to the production of red-pigmented dibenzoquinone oosporein, which is involved in host immune evasion [26].

Two MADS-box transcription factors encoding genes *Bbmcm1* and *Mb1* in *Beauveria bassiana* Bb0062 have been reported to be involved in virulence. Disruption to *Bbmcm1* in *B. bassiana* significantly decreased virulence to *G. mellonella* compared with the wild-type strain. Further studies have found that the deletion of *Bbmcm1* can reduce the production of cuticle-degrading enzyme, which might lead to decreased virulence in the *Bbmcm1*-deletion mutant. Meanwhile, growth, conidiogenesis, and cell integrity in *B. bassiana* could also be influenced by *Bbmcm1* deletion, which also impacts the virulence of *B. bassiana* [27]. In the *Mb1*-deletion mutant of *B. bassiana*, growth and virulence to *G. mellonella* were reduced and cell wall integrity was influenced [28].

Two bZIP transcription factors encoding genes *BbYap1* and *BbHapX* are involved in *Beauveria bassiana* ARSEF 2860 virulence. The deletion of *BbYap1* could impact lipid homeostasis in *B. bassiana*, as well as sensitivity to stress, especially decreasing the virulence to *G. mellonella* compared with the wild-type strain. Deep studies have found that *BbYap1* regulates virulence to insects, mainly through eluding the host humoral defense [29]. The *BbHapX*-deletion mutant influences conidia germination in *B. bassiana* and delays the formation of conidia development into hyphal bodies. In addition, the virulence of *B. bassiana* to *G. mellonella* is reduced in the *BbHapX* disruption mutant compared with the wild-type strain [30].

Transcription factors of the Homeobox, GATA-type, NDT80-like, and p53-like encoding genes *Bbhox2*, *BbAreA*, *Ron1*, and *BbTFO1*, respectively, are involved in *Beauveria bassiana* virulence. The knockout of *Bbhox2* in *B. bassiana* CGMCC7.34 could reduce virulence to *G. mellonella* remarkably compared with the wild-type strain. An in-depth study found that *Bbhox2* was important in appressorium formation and hyphal body development, which is crucial in *B. bassiana* infection. The appressoria and hyphal body were reduced in the *Bbhox2* knockout mutant compared with the wild-type strain [31]. The *BbAreA* mutant of *B. bassiana* decreased the virulence to *G. mellonella* compared with the wild-type strain. Further study found that the production of hyphal bodies was reduced in *BbAreA* knockdown mutants [32]. Disruption to *Ron1* in *B. bassiana* ARSEF 2860 can dramatically reduce virulence to *G. mellonella*. Extracellular chitinase activity is reduced in the *Ron1*-deletion mutant compared with the wild-type strain, which is very important in *B. bassiana* infection. Hyphal bodies are barely visible in the *Ron1*-deletion mutant during infection compared to the wild-type strain, which may be another reason for the decrease in fungal virulence. Moreover, cell wall defects caused by the deletion of *Ron1* could lead *B. bassiana* to adapt environmental stress and may also reduce the virulence of *B. bassiana* [33]. The *BbTFO1*-deletion mutant of *B. bassiana* ARSEF 2860 impacted antioxidant activity, conidial germination, heat stress resistance, and conidial quality. The virulence of the *BbTFO1*-deletion mutant to *G. mellonella* was reduced compared with the wild-type strain. Moreover, the hyphal bodies in the *BbTFO1*-deletion mutant were fewer than that in the wild-type strain, which is important in *B. bassiana* infection [34].

The transcription factor-encoding gene *Bbmsn2* in different *Beauveria bassiana* strains exhibits similar roles in virulence. The deletion of *Bbmsn2* in *B. bassiana* Bb0062 reduced virulence to *Rhipicephalus microplus*, which might be due to the lower protease activity of the *Bbmsn2*-deletion mutant compared with the wild-type and complemented strains during treatment with tick cuticles. The virulence of *B. bassiana* Bb0062 to *G. mellonella* was also impaired after *Bbmsn2* deletion [35]. For the *B. bassiana* strain ARSEF 2860, *Bbmsn2* disruption in *B. bassiana* resulted in significantly reduced virulence to *Spodoptera litura* compared with the wild-type strain. Moreover, the conidia yield and tolerance to stresses of *B. bassiana* ARSEF2860 were also impacted after *Bbmsn2* deletion [36].

Moreover, three HSF transcription factor-encoding genes *hsf1*, *sfl1*, and *skn7*, and two Far/CTF1-type transcription factor-encoding genes *Bbctf1*α and *Bbctf1β*, are involved in *Beauveria bassiana* virulence. The *hsf1*, *sfl1*, and *skn7*-deletion mutants of *B. bassiana* exhibited a remarkably decreased virulence to *G. mellonella*. The deletion of three HSF transcription factors encoding genes also impacted the conidiation, cell wall integrity, and stress tolerance of *B. bassiana* [37]. *Bbctf1α* and *Bbctf1β* were disrupted in *B. bassiana* CICC 41021. Disruption to two genes influenced phenotypic characters, including growth and conidium formation, as well as tolerance to oxidation stress and virulence to *G. mellonella*. The significantly reduced virulence of disruption mutants might be due to the impediment of extracellular lipolytic activities caused by *Bbctf1α* and *Bbctf1β* deletion, which are important in the cuticular penetration of *B. bassiana* by insect pests [38].

The transcription factor-encoding genes *BbpacC* and *Fkh2* positively regulated the virulence of *Beauveria bassiana*. The deletion of *BbpacC* in *B. bassiana* ATCC 90517 resulted in defects in the production of the insecticidal compound dipicolinic acid, and the deletion of *BbpacC* only slightly influenced the virulence of *B. bassiana* to *G. mellonella* and *T. molitor* [39]. *Fkh2* deletion in *B. bassiana* Bb2860 reduced virulence to *G. mellonella*, as well as tolerance to environment stresses, compared with the wild-type strain [40]. The transcription factor-encoding gene *BbStf1* negatively regulated the virulence of *B. bassiana*. The deletion of *BbStf1* in *B. bassiana* Bb0062 increased virulence to *G. mellonella* compared with the wild-type strain. The cell wall integrity and oxidative stress responses were also negatively regulated by *BbStf1*, which is vital in *B. bassiana* infection [41].

### 3.2. Members of the Genus Metarhizium

*Metarhizium* is another commonly used microbial insecticide in agriculture, and several products have been developed for its commercial application [42,43]. Similarly to *B. bassiana*, multiple transcription factors, belonging to different families, including C_2_H_2_-type, APSES-type, GATA-type, Zn_2_Cys_6_, Homeobox, bZIP, GATA-type, and HSF-type, have been reported as being involved in the control of insect pests in *Metarhizium acridum*, *Metarhizium robertsii*, and *Metarhizium rileyi*.

For *Metarhizium acridum*, C_2_H_2_-type is the transcription factor family most reported to be involved in the virulence to insect pests. Five C_2_H_2_-type transcription factors encoding genes *MaMsn2*, *MaPacC*, *MaSte12*, *MaCrz1*, and *MaNCP1*, were reported as being involved in the virulence of *M. acridum* CQMa102. The deletion of *MaMsn2* reduced the virulence of *M. acridum* to *L. migratoria* remarkably [44]. Disruption to *MaPacC* also significantly reduced the virulence of *M. acridum* to *L. migratoria manilensis*. Meanwhile, the deletion of *MaPacC* delayed appressorium formation and impacted the expression of some insect cuticle hydrolases, which may be a reason for the decreased virulence [45]. Similarly, *MaSte12* deletion in *M. acridum* significantly reduced the virulence to *L. migratoria*. Further studies showed that *MaSte12* deletion could influence appressorium formation, as well as penetration, in *M. acridum* [46]. *MaCrz1* disruption in *M. acridum* caused decreased virulence to *L. migratoria manilensis*, as well as impaired the penetration ability in the host cuticle in the mutant. Meanwhile, tolerance to stress and cell walls were also affected in the *MaCrz1*-deletion mutant, which may be another factor influencing virulence. Additionally, cuticle-degrading genes were associated with virulence in *M. acridum* [47]. *MaNCP1* deletion in *M. acridum* influenced cuticular penetration ability and decrease virulence to *L. migratoria manilensis*. Further studies found that the expression levels of cuticle-degrading genes were downregulated after *MaNCP1* deletion [48].

The GATA-type and Zn(II)_2_Cys_6_ transcription factors encoding genes *MaAreB* and *MaAzaR* were associated with virulence in *Metarhizium acridum* CQMa102. The deletion of *MaAreB* decreased the virulence of *M. acridum* to locusts, which may be due to defects in appressorium formation and appressorial turgor pressure after *MaAreB* deletion. In addition, the cell wall integrity was also affected in the *MaAreB*-deletion mutant [49]. *MaAzaR* deletion also decreased the virulence of *M. acridum* to *L. migratoria*. The decreased virulence in the *MaAzaR*-deletion mutant is mainly due to defects in appressoria, including appressorium formation, appressorial turgor pressure, and hydrolytic enzymes compared with the wild-type strain [50].

Two other transcription factors encoding genes *MaFTF1* and *MaSom1* were related to virulence in *Metarhizium acridum* CQMa102. The overexpression of *MaSom1* enhanced the virulence of *M. acridum* to *L. migratoria manilensis*. Besides the virulence to insect pests, stress tolerance and conidiation were also altered in *M. acridum* after *MaSom1* overexpression [51], while *MaFTF1* negatively regulated the virulence of *M. acridum*. The knockout of *MaFTF1* caused increased virulence in *M. acridum* to *Locusta migratoria manilensis*. An in-depth study found that the deletion of *MaFTF1* accelerated the development of appressoria, together with higher appressorial turgor pressure, compared with the wild-type strain [52].

In *Metarhizium rileyi*, the C_2_H_2_-type family is the transcription factor family most reported as being involved in the virulence to insect pests. Three C_2_H_2_-type transcription factors encoding genes *MripacC*, *MrSte12*, and *MrMsn2* have been reported as being involved in the virulence of *M. rileyi* CQNr01. The virulence of *M. rileyi* to *S. litura* was reduced after the deletion of *MripacC*. Deep study found that the conidium surface structure was smoother in the *MripacC*-deletion mutant compared with the wild-type strain, which is important in the conidia-adhesion ability of *M. rileyi*. Moreover, the ability to secrete protein-degrading enzymes was decreased after *MripacC* deletion, which might be the reason for the decreased virulence of the *MripacC*-deletion mutant [53]. *MrSte12* deletion significantly reduced the virulence of *M. rileyi* to *S. litura*. The decreased virulence in the *MrSte12*-deletion mutant may be due to impaired appressorium formation after *MrSte12* deletion. Moreover, the deletion of *MrSte12* also influenced growth, conidiation, and tolerance to stress [54]. The deletion of *MrMsn2* also decreased virulence of *M. rileyi* to *S. litura*, as well as microsclerotia formation, remarkably [55].

Besides C_2_H_2_-type transcription factors, genes encoding bZIP (*Mrap1*), GATA-type (*MrNsdD*), and APSES-type (*MrStuA* and *MrXbp*) transcription factors are involved in the virulence of *Metarhizium rileyi* CQNr01. The deletion of *Mrap1* in *M. rileyi* decreased the virulence to *S. litura*, as well as conidial and microsclerotial yield, compared with the wild-type strain [56]. *MrNsdD* disruption in *M. rileyi* affected virulence to *S. litura*, as well as microsclerotium formation and conidiation in the *MrNsdD*-deletion mutant compared with the wild-type strain [57]. *M. rileyi* mutants with the deletion of *MrStuA* and *MrXbp*, respectively, exhibited impaired virulence to *S. litura*, as well as microsclerotium formation, conidiation, and stress tolerance compared with the wild-type strain [58]. Similarly, the deletion of the transcription factor-encoding gene MrSwi6 in *M. rileyi* resulted in reduced virulence toward *S. litura*, as well as conidiation and microsclerotia formation compared with the wild-type and complemented strains [59].

In *Metarhizium robertsii*, three different transcription factor families, including genes encoding bZIP (*MBZ1*), C_2_H_2_-type (*MrpacC*), and Zn_2_Cys_6_ (*Aftf1*), have been found to be related to virulence in *M. robertsii* ARSEF 2575. The deletion of *MrpacC* impacted the virulence of *M. robertsii* to *Bombyx mori*, as well as reduced chitinase activity, compared with the wild-type strain. The impaired virulence in the *MrpacC*-deletion mutant may be due to *MrpacC* deletion influencing the cuticle-penetration ability [60]. The *MBZ1*-deletion mutant exhibited impaired virulence to *B.mori* and *G. mellonella*. Moreover, growth, conidiogenesis, and cell wall integrity were influenced by *MBZ1* deletion in *M. robertsii* [61]. The *Aftf1*-disruption mutant also showed remarkably reduced virulence to *G. mellonella*, as well as delayed appressorial formation, compared with the wild-type strain [62].

Genes encoding three types of transcription factors, homeobox (*MrHOX7*), HSF (*MrSkn7*), and APSES (*MrStuA*), are involved in virulence in *Metarhizium robertsii* ARSEF 23. The *MrHOX7*-disruption mutant shows inhibited virulence to *G. mellonella.* An in-depth study found that appressorium formation and conidial adhesion were reduced and the expression of adhesion- and appressorium-related encoding genes were downregulated in the *MrHOX7*-disruption mutant compared with the wild-type strain, which may be the reason behind the inhibited virulence after *MrHOX7* disruption [63]. The deletion of *MrStuA* and *MrSkn7* reduced virulence to *G. mellonella*. In addition, appressorium-formation ability was lost after *MrSkn7* deletion compared with the wild-type strain, which may be a reason for the reduced virulence in the *MrSkn7*-deletion mutant [64,65].

Two other transcription factors encoding genes *MrSt12* and *Mrmsn2* have been associated with virulence in *Metarhizium robertsii* ARSEF 2575. Disruption to *MrSt12* causes *M. robertsii* to lose virulence to *G. mellonella*, which may be due to the appressoria not being produced in the *MrSt12*-deletion mutant compared with the wild-type and complemented strains [66]. The *Mrmsn2*-deletion mutant showed remarkably reduced virulence to *T. molitor* compared with the wild-type strain. In addition, conidiation and tolerance to environmental stresses were influenced by *Mrmsn2* deletion [36].

### 3.3. Arthrobotrys Oligospora, Arthrobotrys Flagrans, and Drechslerella Dactyloides

*Arthrobotrys oligospora* and *Arthrobotrys flagrans* are nematode-trapping fungi that capture plant-pathogenic nematodes by producing specialized trap structures [67,68]. Several different types of transcription factors have been reported as being involved in the pathogenesis of *Arthrobotrys* sp. Two C_2_H_2_-type transcription factors encoding genes *AoMsn2* and *AoSte12* are deleted in *A. oligospora* ATCC 24927. Both deletion mutants influence pathogenicity to *Caenorhabditis elegans*. Deep study found that the deletion of *AoMsn2* significantly decreased the number of traps compared with the wild-type strain [69]. In the *AoSte12* mutant, both the hyphal ring traps and electron-dense bodies were increased compared with the wild-type strain [70].

Disruption to the genes encoding two other types of transcription factors, MADS-box (*AoRlmA*) and APSES (*AoStuA*), in *Arthrobotrys oligospora* ATCC 24927, also impacted pathogenicity to *C. elegans*. In the *AoRlmA*-disruption mutant, growth, sporulation, trap formation, and stress tolerance were also influenced compared with the wild-type strain [71]. The *AoStuA*-deletion mutant could not form traps and thereby lost the ability to capture *C. elegans*. Meanwhile, proteolytic activity was decreased after *AoStuA* deletion [72].

Besides *Arthrobotrys oligospora*, transcription factors in *Arthrobotrys flagrans* have also been reported to be involved in pathogenicity to nematodes. The knockout of the APSES transcription factor-encoding gene *AfSwi6* impacted the pathogenicity of *A. flagrans* YMF1.07536 to *C. elegans*. The number of traps was reduced after *AfSwi6* deletion. In addition, the extracellular protease activity was remarkably reduced in the mutant compared with the wild-type strain [73].

*Drechslerella dactyloides* is another type of nematode-trapping fungi used to control nematodes [74]. The deletion of the C_2_H_2_-type transcription factor gene *DdaCrz1* in *D. dactyloides* 29 resulted in fewer traps being formed and lower constricting ring inflation compared with the wild-type strain after the introduction of *C. elegans*. In addition, growth, conidiation, stress tolerance, and cell wall integrity were influenced by *DdaCrz1* deletion [75]. Similarly, with the deletion of another C_2_H_2_-type transcription factor gene, *DdaSTE12*, in *D. dactyloides* 29, trap formation and ring cell inflation were also decreased compared with the wild-type strain after the introduction of *C. elegans* [76].

### 3.4. Members of the Genus Trichoderma

*Trichoderma* is an important mycoparasite agent that is used widely to control fungal plant pathogens such as *Rhizoctonia solani*, *Sclerotinia sclerotiorum*, *Botrytis cinerea,* and *Fusarium oxysporum* [77,78,79]. Several transcription factors, belonging to different families, such as C_2_H_2_-type, GATA-type, and MYB, have been reported as being involved in the biocontrol of *T. atroviride*, *T. asperellum*, and *T. harzianum*.

In *Trichoderma harzianum*, transcription factor genes belonging to C_2_H_2_-type (*Tha09974*), C6 zinc finger (*Thc6*), and Cys_6_Zn(II)_2_ (*Thctf1*) have been associated with the biocontrol potential of *T. harzianum* toward pathogens. The deletion of *Tha09974* in *T. harzianum* Th33 could reduce its antagonistic ability toward *B. cinerea* and *F. oxysporum* compared with the wild-type strain. *Tha09974* deletion could also influence the biomass and spore production of *T. harzianum* [80]. Knockout and overexpression were performed to analyze the role of *Thc6* in *T. harzianum* Th22 against *Curvularia lunata*. Compared with the wild-type strain, the overexpression mutant reduced the disease index caused by *C. lunata* to maize, while the knockout mutant increased the disease index [81]. The *Thctf1* disruption mutant of *T. harzianum* CECT 2413 showed a significantly reduced antifungal ability toward *B. cinerea*. The production of 2-pentyl furan and benzaldehyde, which are antifungal volatiles, was influenced by *Thctf1* deletion [82].

Two other transcription factors, *pac1* and *ThpacC*, have also been related to the antagonistic ability of *Trichoderma harzianum*. The mutation of *pac1* in *T. harzianum* CECT 2413 was performed through RNA interference. The antagonistic ability of the mutant toward *R. solani*, *R. meloni*, and *Phytophthora citrophthora* was reduced compared with the wild-type strain. As well as the expression of genes involved in antagonism, such as chit42, chitinase was reduced compared with the wild-type strain [83]. The knockout of *ThpacC* in *T. harzianum* 3.9236 could reduce the antagonistic ability toward *S. sclerotiorum* compared with the wild-type strain and overexpression mutant. An in-depth study found that the production of two antifungal compounds, homodimericin A and 8-*epi*-homodimericin A, were abolished after knockout, which may be the reason behind the reduced antagonistic ability of the *ThpacC*-deletion mutant [84].

A C_2_H_2_-type transcription factor-encoding gene *Ste12*, and a GATA-type transcription factor-encoding gene *Are1*, are involved in the mycoparasitic ability of *Trichoderma atroviride* P1. Disruption to *Ste12* and *Are1* could reduce the mycoparasitic ability of *T. atroviride* P1 to *R. solani* and *B. cinerea*, respectively, compared with the wild type [85]. Moreover, diffusible metabolites from the *Are1* deletion mutant exhibit reduced antifungal capacity compared with the wild-type strain [86].

*Trichoderma asperellum* and *Trichoderma virens* are also transcription factors that have been reported as being involved in the biocontrol of pathogens. The role of the MYB transcription factor-encoding gene *MYB36* in *T. asperellum* Tas653 against *Alternaria alternata* was analyzed through gene knockout and overexpression. Compared with the wild-type strain, the *MYB36*-knockout mutant reduced the inhibition ability against *A. alternata*, while the *MYB36*-overexpression mutant improved the hyperparasitic ability. In addition, the activities of superoxide dismutase, peroxidase, and catalase were also reduced after *MYB36* deletion [87]. The deletion of the transcription factor-encoding gene *pacC* in *T. virens* IMI 304061 reduced the biocontrol potential toward *R. solani* and *S. rolfsii*, which might be due to *pacC* deletion impairing the adaptability of *T. virens* to an alkaline pH or its capacity to perceive ambient pH [88].

### 3.5. Clonostachys Rosea and Coniothyrium Minitans

Similarly to *Trichoderma*, *Clonostachys rosea* is a very important biocontrol mycoparasite that can control numerous fungal plant pathogens [89,90]. The bHLH transcription factor-encoding gene *sre1* plays different regulation roles in *C. rosea* IK726 antagonism. The deletion of *sre1* improved the antagonistic ability toward *B. cinerea* but led to a decreased antagonistic ability to *R. solani*. A further study found that the expression of antagonism-related genes such as polyketide synthases and chitinases were downregulated in the *sre1*-deletion mutant compared with the wild-type strain [91]. The deletion of the transcription factor-encoding gene *pacC* in *C. rosea* 611 could significantly decrease its virulence to *Panagrellus redivivus*. The expression level of extracellular serine protease in the *pacC*-deletion mutant was downregulated [92]. *crtf* is a gene encoding the Tubby transcription factor; the *crtf*-deletion mutant of *C. chloroleuca* 67-1 reduced its parasitic ability of sclerotia in *S. sclerotiorum* and significantly lowered the biocontrol capacity toward soybean Sclerotinia white mold caused by *S. sclerotiorum* [93].

Similarly to *Trichoderma* and *Clonostachys*, *Coniothyrium minitans* is commonly used as a biocontrol agent to control plant disease [94,95]. Disruption to the transcription factor-encoding gene *CmpacC* in *C. minitans* Chy-1 remarkably reduces mycoparasitic ability to sclerotia in *S. sclerotiorum*. The production of the mycoparasitism-related enzymes chitinase and β-1,3-glucanse is notably suppressed in the *CmpacC*-deletion mutant compared with the wild-type and complemented strains [96]. The NDT80-like transcription factor-encoding gene *CmNdt80a* in *C. minitans* ZS-1 has been found to play a similar role in mycoparasitic ability. The deletion of *CmNdt80a* in *C. minitans* ZS-1 significantly reduced the parasitic ability to sclerotia of *S. sclerotiorum*. The cell wall integrity and conidiation were also affected by *CmNdt80a* deletion [97].

### 3.6. Other Biocontrol Fungi

Transcription factors from other biocontrol fungi, including *Hirsutella minnesotensis*, *Candida oleophila*, *Papiliotrema terrestris*, *Paecilomyces lilacinus* (synonyms of *Purpureocillium lilacinum*) and *Purpureocillium lilacinum*, have been reported as being involved in biocontrol. The deletion of the Zn(II)_2_Cys_6_ transcription factor-encoding gene *rolP* in *P. lilacinus* Pl36-1 could significantly impact its pathogenicity to *Meloidogyne incognita*. Moreover, the nematotoxin leucinostatin A was absent from the *rolP*-deletion mutant compared with the wild-type strain [98]. Disruption of the HSF transcription factor-encoding gene HIM-SKN7 in *Hirsutella minnesotensis* 3608 could reduce its endoparasitic ability to *Heterodera glycines* compared with the wild-type and overexpression strains. Moreover, conidiation and stress tolerance were affected after HIM-SKN7 deletion [99].

Two bZIP transcription factor-encoding genes *Yap1* and *lcsL*, have been disrupted in *Papiliotrema terrestris* and *Purpureocillium lilacinum*, respectively. *Yap1* deletion in *P. terrestris* LS28 decreased its biocontrol activity against *Penicillium expansum* and *Monilinia fructigena* [100]. *lcsL* was disrupted in *Purpureocillium lilacinum* PLBJ-1. The *lcsL*-disruption mutant lost its antagonistic ability to *Phytophthora infestans*; moreover, no leucinostatins were detected, in contrast to the wild-type and overexpression strains [101]. Besides the two bZIP transcription factors, the MADS-box transcription factor-encoding gene *Rlm1* has also been found to be involved in biocontrol. The deletion of the MADS-box transcription factor-encoding gene *Rlm1* in *C. oleophila* I-182 resulted in decreased biocontrol efficacy to gray mold on kiwifruit caused by *B. cinerea* compared with the wild-type strain. In addition, the deletion of *Rlm1* also influenced tolerance to stresses [102].

## 4. Conclusions and Future Prospects

Transcription factors are involved in the regulation of multiple biological processes, including fungal growth, development, morphological characteristics, cell wall integrity, tolerance to stresses, virulence, conidiation, and the production of secondary metabolites. Biocontrol is a complex biological process that, mainly through the production of cell wall-degrading enzymes, toxins, and/or antibiotics, exerts activity using biocontrol fungi. Many of these biocontrol behaviors are regulated by transcription factors. Therefore, we investigated the roles of transcription factors in biocontrol and the corresponding regulation mechanisms. The presented studies may be useful for further analyses of the molecular mechanisms underlying biocontrol and in improving the control efficiency of biocontrol fungi. At present, the transcription factors reported as being involved in biocontrol belong to the families C_2_H_2_, bZIP, GATA, Zn_2_Cys_6_, MADS-box, HSF, APSES, and Homeobox. To date, biocontrol-related transcription factors have been reported in biocontrol fungi including *Beauveria bassiana*, *Metarhizium acridum*, *M. robertsii*, *M. rileyi*, *Arthrobotrys oligospora*, *A. flagrans*, *Drechslerella dactyloides*, *Clonostachys rosea*, *Coniothyrium minitans*, *Trichoderma harzianum*, *T. asperellum*, *T. virens*, and *T. atroviride,* which have been used to control insect pests, plant pathogenic nematodes, and fungal plant pathogens. Although numerous studies on biocontrol transcription factors have been reported, systematic and comprehensive reviews summarizing and analyzing transcription factors in biocontrol fungi are rare. Therefore, this review mainly introduced and discussed the roles of transcription factors in biocontrol fungi, as well as the potential underlying biocontrol mechanisms. This review provides a basis for the further elaboration of the molecular mechanisms behind biocontrol and further improvements to control efficiency in biocontrol fungi.

In biocontrol fungi, transcription factors were involved in regulation of the physiological characteristics of fungus, including growth, conidial development, spore germination, microsclerotium formation, cell wall integrity, stress tolerance, formation of appressorium, traps, and hyphal body. As well as biochemical traits containing activities of cell wall-degrading enzymes of cuticle-degrading proteases, chitinase and β-1,3-glucanse; the activities of superoxide dismutase, peroxidase, and catalase; and production of secondary metabolites of homodimericin A, 8-epi-homodimericin A, 2-pentyl furan and benzaldehyde and nematotoxin leucinostatin A were also regulated by transcription factors of biocontrol fungus. Moreover, the expression of genes involved in biocontrol, including genes encoding serine protease, chitinases, adhesion- and appressorium-related, polyketide synthases, were affected after transcription factor disruption.

Future studies should focus on the development of more transcription factors for use in biocontrol, the construction of gene-engineered stains through the overexpression of biocontrol-related transcription factors encoding genes to improve control efficacy, and the elucidation of regulation networks of transcription factors that are active during the processes of biocontrol.

(1) Developing more biocontrol-related transcription factors: Compared with the currently reported biocontrol fungal species, there are fewer corresponding types of transcription factors involved in biocontrol. There are many types of biocontrol fungi in which no biocontrol transcription factors have been reported. Moreover, at present, among the total transcription factor families, the reported transcription factor families related to biocontrol only cover a small proportion. Developing more types of transcription factors from more families will be useful in comprehensively elaborating the mechanisms underlying the regulation of biocontrol using transcription factors.
(2) The construction of transcription factor engineering strains: The overexpression of biocontrol-related transcription factors encoding genes in the same biocontrol fungi or the expression of the above genes in different biocontrol fungi can be useful to improve control efficacy.
(3) The screening of transcription factors upstream of regulated genes and downstream of target genes and the analysis of the roles of these genes in biocontrol: This will allow for the construction of a regulation network of biocontrol-related transcription factors and a comprehensive exploration of the molecular mechanisms behind the regulation of biocontrol using transcription factors.

## Figures and Tables

**Table 1:** Transcription factors in biocontrol fungi.

Biocontrol Fungus	Transcription Factors	Family	Pathogens	Reference
*Beauveria bassiana*	*BbYap1*	bZIP	*Galleria mellonella*	29
	*BbCDR1*	Zn_2_Cys_6_	*Galleria mellonella*	21
	*BbSmr1*	C_2_H_2_-type	*Galleria mellonella*	26
	*Bbotf1*	Zn(II)_2_Cys_6_	*Galleria mellonella*	19
	*Bbhox2*	Homeobox	*Galleria mellonella*	31
	*Bbklf1*	C_2_H_2_-type	*Galleria mellonella*	23
	*Ron1*	NDT80	*Galleria mellonella*	33
	*Mb1*	MADS-box	*Galleria mellonella*	28
	*Bbctf1α*	Far/CTF1-type	*Galleria mellonella*	38
	*Bbctf1β*	Far/CTF1-type	*Galleria mellonella*	38
	*BbHapX*	bZIP	*Galleria mellonella*	30
	*BbTpc1*	Zn(II)_2_Cys_6_	*Galleria mellonella*	18
	*BbStf1*	Leucine zipper dimerization	*Galleria mellonella*	41
	*BbTFO1*	p53-like	*Galleria mellonella*	34
	*Bbmsn2*	—	*Galleria mellonella*; *Spodoptera litura*; *Rhipicephalus microplus*	35, 36
	*BbThm1*	Zn(II)_2_Cys_6_	*Galleria mellonella*	20
	*Bbmcm1*	MADS-box	*Galleria mellonella*	27
	*Crz1*	C_2_H_2_-type	*Spodoptera litura*	24
	*BbPacC*	—	*Galleria mellonella*; *Tenebrio molitor*	39
	*zafa*	C_2_H_2_-type	*Galleria mellonella*	25
	*NirA1*	Zn_2_Cys_6_	*Galleria mellonella*	22
	*Fkh2*	—	*Galleria mellonella*	40
	*BbAreA*	GATA-type	*Galleria mellonella*	32
	*hsf1*	HSF-type	*Galleria mellonella*	37
	*skn7*	HSF-type	*Galleria mellonella*	37
	*sfl1*	HSF-type	*Galleria mellonella*	37
*Metarhizium acridum*	*MaFTF1*	—	*Locusta migratoria manilensis*	52
	*MaAzaR*	Zn(II)_2_Cys_6_	*Locusta migratoria manilensis*	50
	*MaSom1*	—	*Locusta migratoria manilensis*	51
	*MaAreB*	GATA-type	Locust	49
	*MaPacC*	C_2_H_2_-type	*Locusta migratoria manilensis*	45
	*MaSte12*	C_2_H_2_-type	*Locusta migratoria*	46
	*MaCrz1*	C_2_H_2_-type	*Locusta migratoria manilensis*	47
	*MaMsn2*	C_2_H_2_-type	*Locusta migratoria*	44
	*MaNCP1*	C_2_H_2_-type	*Locusta migratoria manilensis*	48
*Metarhizium robertsii*	*MrHOX7*	Homeobox	*Galleria mellonella*	63
	*MrSt12*	—	*Galleria mellonella*	66
	*MrStuA*	APSES-type	*Galleria mellonella*	58
	*MrSkn7*	HSF-type	*Galleria mellonella*	65
	*MBZ1*	bZIP	*Galleria mellonella*; *Bombyx mori*	61
	*MrpacC*	C_2_H_2_-type	*Bombyx mori*	60
	*Mrmsn2*	—	*Tenebrio molitor*	36
	*Aftf1*	Zn_2_Cys_6_	*Galleria mellonella*	62
*Metarhizium rileyi*	*MrSte12*	C_2_H_2_-type	*Spodoptera litura*	54
	*MrNsdD*	GATA-type	*Spodoptera litura*	57
	*MrStuA*	APSES-type	*Spodoptera litura*	64
	*MrXbp*	APSES-type	*Spodoptera litura*	58
	*MrSwi6*	—	*Spodoptera litura*	59
	*MrMsn2*	C_2_H_2_-type	*Spodoptera litura*	36
	*Mrap1*	bZIP	*Spodoptera litura*	56
	*MripacC*	C_2_H_2_-type	*Spodoptera litura*	53
*Coniothyrium minitans*	*CmNdt80a*	NDT80	*Sclerotinia sclerotiorum*	97
	*CmpacC*	—	*Sclerotinia sclerotiorum*	96
*Clonostachys rosea*	*pacC*	—	*Panagrellus redivivus*	92
	*sre1*	bHLH	*Botrytis cinerea*; *Rhizoctonia solani*	91
*Clonostachys chloroleuca*	*crtf*	Tubby	*Sclerotinia sclerotiorum*	93
*Trichoderma harzianum*	*ThpacC*	—	*Sclerotinia sclerotiorum*	84
	*Thctf1*	Cys_6_Zn(II)_2_	*Botrytis cinerea*	82
	*pac1*	—	*Rhizoctonia solani*; *Rhizoctonia meloni*; *Phytophthora citrophthora*	83
	*Thc6*	C6 zinc finger	*Curvularia lunata*	81
	*Tha09974*	C_2_H_2_-type	*Botrytis cinerea*; *Fusarium oxysporum*	80
*Trichoderma asperellum*	*MYB36*	MYB	*Alternaria alternata*	87
*Trichoderma virens*	*pacC*	—	*Rhizoctonia solani*; *Sclerotium rolfsii*	88
*Trichoderma atroviride*	*Ste12*	C_2_H_2_-type	*Rhizoctonia solani*; *Botrytis cinerea*	85
	*are1*	GATA-type	*Rhizoctonia solani*; *Botrytis cinerea*	86
*Hirsutella minnesotensis*	*HIM-SKN7*	HSF-type	*Heterodera glycines*	99
*Candida oleophila*	*Rlm1*	MADS-box	*Botrytis cinerea*	102
*Arthrobotrys flagrans*	*AfSwi6*	APSES-type	*Caenorhabditis elegans*	73
*Arthrobotrys oligospora*	*Aomsn2*	C_2_H_2_-type	*Caenorhabditis elegans*	69
	*AoRlmA*	MADS-box	*Caenorhabditis elegans*	71
	*AoStuA*	APSES-type	*Caenorhabditis elegans*	72
	*AoSte12*	C_2_H_2_-type	*Caenorhabditis elegans*	70
*Drechslerella dactyloides*	*DdaCrz1*	C_2_H_2_-type	*Caenorhabditis elegans*	75
	*DdaSTE12*	C_2_H_2_-type	*Caenorhabditis elegans*	76
*Paecilomyces lilacinus*	*rolP*	Zn(II)_2_Cys_6_	*Meloidogyne incognita*	98
*Purpureocillium lilacinum*	*lcsL*	bZIP	*Phytophthora infestans*	101
*Papiliotrema terrestris*	*yap1*	bZIP	*Penicillium expansum*; *Monilinia fructigena*	100

## Data Availability

Not applicable.
